# Analysis on Four Derivative Waveforms of Photoplethysmogram (PPG) for Fiducial Point Detection

**DOI:** 10.3389/fpubh.2022.920946

**Published:** 2022-06-30

**Authors:** Mohd Zubir Suboh, Rosmina Jaafar, Nazrul Anuar Nayan, Noor Hasmiza Harun, Mohd Shawal Faizal Mohamad

**Affiliations:** ^1^Faculty of Engineering and Built Environment, Universiti Kebangsaan Malaysia, Bangi, Malaysia; ^2^Medical Engineering Technology Section, British Malaysian Institute, Universiti Kuala Lumpur, Kuala Lumpur, Malaysia; ^3^Department of Medicine, Hospital Canselor Tuanku Muhriz, Kuala Lumpur, Malaysia

**Keywords:** acceleration plethysmogram (APG), photoplethysmogram (PPG), PPG derivatives, PPG fiducial points, velocity plethysmogram (VPG)

## Abstract

Fiducial points of photoplethysmogram (PPG), first derivative PPG (VPG), and second derivative PPG (APG) are essential in extracting numerous parameters to diagnose cardiovascular disease. However, the fiducial points were usually detected using complex mathematical algorithms. Inflection points from derivatives waveforms are not thoroughly studied, whereas they can significantly assist in peak detection. This study is performed to investigate the derivative waveforms of PPG and use them to detect the important peaks of PPG, VPG, and APG. PPGs with different morphologies from 43 ischemic heart disease subjects are analyzed. Inflection points of the derivative waveforms up to the fourth level are observed, and consistent information (derivative markers) is used to detect the fiducial points of PPG, VPG, and APG with proper sequence. Moving average filter and simple thresholding techniques are applied to detect the primary points in VPG and the third derivative waveform. A total of twelve out of twenty derivative markers are found reliable in detecting fiducial points of two common types of PPG. Systolic peaks are accurately detected with 99.64% sensitivity and 99.38% positive predictivity using the 43 IHD dataset and Complex System Laboratory (CSL) Pulse Oximetry Artifact Labels database. The study has introduced the fourth derivative PPG waveform with four main points, which are significantly valuable for detecting the fiducial points of PPG, VPG, and APG.

## Introduction

The simple electro-optical technique of photoplethysmography is used to detect the blood volume changes in vascular tissue beds at peripheral parts of the body, such as fingertip, earlobe, and toe. This technique is clinically used to monitor heart rate and oxygen saturation level in the blood, which are extracted from the pulsatile waveform known as photoplethysmogram (PPG) (1). PPG is preferable to other non-invasive techniques such as electrocardiography (ECG) and phonocardiography (PCG) for disease diagnosis since its technology is simple, portable, and inexpensive and does not require an expert to operate. These advantages are good for real-time monitoring of a patient (2).

Photoplethysmogram waveform has been widely studied to diagnose cardiovascular disease (CVD). Various CVD-related parameters can be obtained from PPG. These include heart rate (HR), heart rate variability (3), respiration rate (4), estimation of blood pressure (5), vascular aging index, ankle-brachial pressure index, and pulse transit time that has a strong correlation with arterial stiffness (5, 6). Parameters other than those specified can be found in Ab Hamid and Nayan (7). Evaluation and selection of these parameters are made as features for disease prediction or classification (8).

Photoplethysmogram parameters are obtained from time-series information of PPG, first derivative PPG, and second derivatives of PPG waveforms. The first derivative PPG is also known as velocity plethysmogram (VPG), whereas the second derivative is called acceleration plethysmogram (APG). The typical fiducial points for PPG, VPG, and APG are as follows:

- PPG: onset, systolic, notch, and diastolic peaks
- VPG: u, v, and w peaks
- APG: a, b, c, d, and e peaks

Photoplethysmogram is recorded using a pulse oximetry device, which is sensitive to skin structure (color), skin temperature, electrical noise, motion artifacts, and measuring environment (9). PPG is also significantly affected by the physiological variability of aging, hypertension, diabetes, vessel compliance, and apnoeic episodes (10). These have caused the peak detection of PPG to become challenging. Multiple methods to detect systolic peaks have been reported using a few filters or rules (9, 10) and involve complex mathematical algorithms (6, 11, 12). Meanwhile, there is also a simple method to detect the peak using the derivatives method as reported in Li et al. (13) and Elgendi et al. (14), where the systolic peak can be easily detected using the zero-crossing point after the maximum amplitude of VPG.

The information on such marker (derivative marker) is helpful for PPG peak detection as the derivative highlights the turning points of a signal based on the slope of the original signal. Thus, this study is performed to identify consistent markers obtained from PPG and its derivative waveforms and use them to detect all PPG, VPG, and APG peaks. Direct information from the inflection points from the derivatives signals (derivatives markers) is used without the need to generate a block of interest for systolic and notch peak locations as previously proposed by Li et al. (13) and Elgendi et al. (14), respectively. A set of 43 PPG data is recorded from newly diagnosed ischemic heart disease (IHD) subjects to get various morphology of PPG waveforms for analysis. The performance of the proposed method is evaluated using the 43 IHD datasets and Complex System Laboratory (CSL) Pulse Oximetry Artifact Labels database.

## PPG and PPG Derivatives

**Figure 1:**
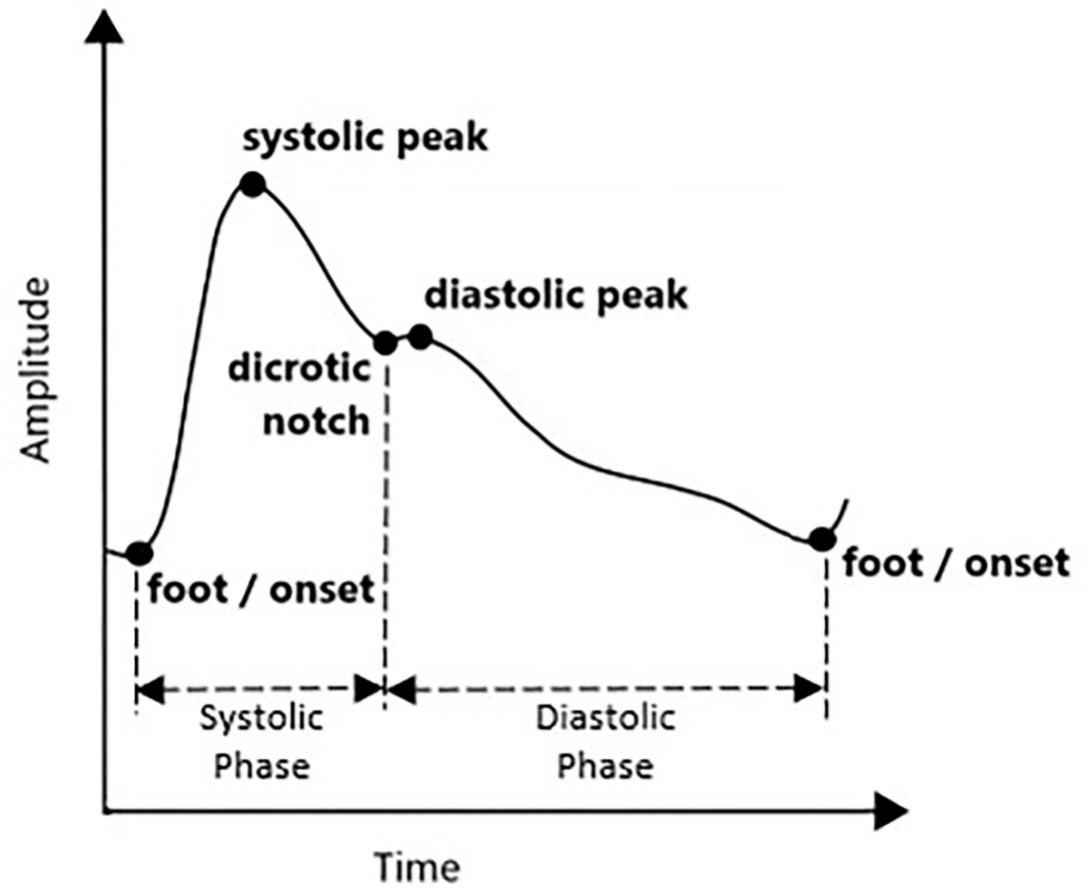
Typical PPG waveform.

Photoplethysmogram waveform comes with four main fiducial points of onset (foot), systolic peak, dicrotic notch, and diastolic peak. This is shown in Figure 1. Onset denotes the beginning of the pulse in the systolic phase, where blood with oxygen flows at the measuring site. The systolic peak is the maximum peak following the onset during systolic ejection. The rising edge from onset to systolic represents increasing pressure in the artery, as measured in the arterial blood pressure (ABP) waveform. The dicrotic notch and diastolic peaks are wave reflections of the periphery, influenced by arterial stiffness or vascular resistance and compliance (15). If onset is related to the beginning of systolic ejection, then diastolic denotes the end of ejection as the aortic valve closed (16). As people start aging, the notch and diastolic tend to disappear in the signal (15).

Detection of onset and systolic locations are commonly reported in previous studies. In general, a pulse's minimum and maximum amplitude points are considered onset and systolic, respectively. This method is applied in Shin et al. (17) with the adaptive thresholding technique. However, threshold-dependent techniques are limited to low amplitude and noisy signals (11). Multiple other methods are explored to highlight the systolic peak, such as Shannon energy, Hilbert transform, variational mode decomposition, mountaineer's method, numerous filters, and wavelet methods (11, 18–21). Elgendi (9), and Chakraborty et al. (10), Li et al. (13), and Kazanavicius and Gircys (22) have successfully utilized the PPG derivative information to detect the fiducial points of PPG. According to Elgendi (9), derivatives are helpful since they allow PPG signals to be interpreted easily by recognizing multiple inflection points.

Velocity plethysmogram reflects the velocity of the amplitude changes in time series and contains three prominent peaks of maximum slope point in systolic (u-peak), minimum slope point in diastolic (v-peak), and maximum slope point in diastolic (w-peak) (23). Zero-crossing points before and after u-peak are used in Li et al. (13) to find onset and systolic peak. The zero-crossing point after w-peak is equivalent to the diastolic peak. It is the changes between w-peak and u-peak (time taken from systolic to diastolic peak), which is used to measure the stiffness index of an artery (24). However, there are cases when amplitude w does not exceed zero, and multiple low amplitude positive waves appear in the diastolic phase. This might be related to electrical noise, dc offset, or filtering offset on the raw PPG signal. Therefore, this derivative marker is rarely used.

**Figure 2:**
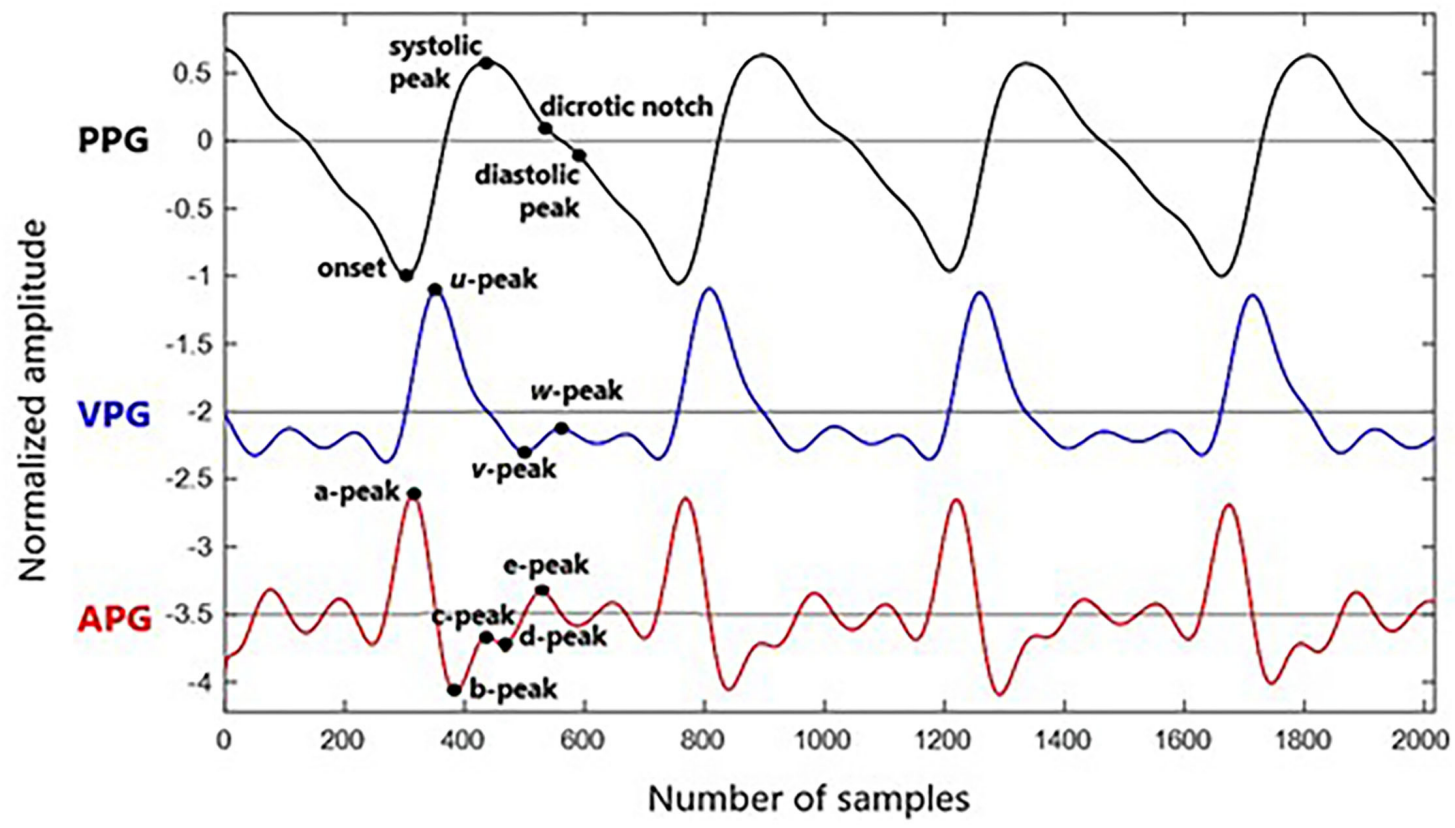
Fiducial points of PPG, VPG, and APG waveform.

Analysis of APG is more frequent compared to VPG. In APG waveform, there are four waves in the systolic phase of a, b, c, and d as well as one early-diastolic e-wave. One crucial marker here is that the e-peak is equivalent to a dicrotic notch. Then, diastolic peak can be determined through the first local maxima after the notch (25). However, aging and other pathological factors that influence the waveform can complicate the detection of diastolic peaks through this method. Another method of estimating the diastolic notch by examining the VPG has been proposed in Millasseau et al. (24). Example waveforms of synchronized PPG, VPG, and APG with their fiducial points are shown in Figure 2.

Besides APG and VPG, the third derivative of PPG has also been explored. Charton et al. have identified two fiducial points of p1-peak and p2-peak (corresponds to early and late systolic components) and used them to extract more features in assessing mental stress (25). The information on each fiducial point from each PPG derivative is dispersed in many publications, and they are not fully utilized for PPG peak detection.

Detected locations of the fiducial points are translated to time and amplitude for analysis. The slope and area of each wave are normally studied as well, in differentiating between healthy and unhealthy subjects. Previous studies have discovered a few important parameters utilizing the fiducial points of PPG and APG for CVD diagnosis. The parameters are summarized in Methodology in Table 1.

**Table 1:** Significant CVD-related predictors based on PPG fiducial points.

**Signal**	**PPG Parameters**	**Measurement**	**Remarks**	**Significance**
PPG	Stiffness Index (SI)	$SI=\frac{H}{DT}$	H = Subject height; DT, Delta Time (Time from systolic peak to diastolic peak).	Risk factor for coronary heart diseases such as hypertension, diabetes, and smoking is proportional to the stiffness index (26).
PPG	Reflection Index (RI)	$RI=\frac{D_{peak}}{S_{peak}}\times 100\%$	D_peak_ = Amplitude of diastolic peak from the onset. S_peak_ = Amplitude of systolic peak from the onset.	A good indicator for vascular assessment (27).
PPG	Augmentation Index (AIx)	$SI=\frac{S_{peak}-D_{peak}}{S_{peak}}\times 100\%$	D_peak_ = Amplitude of diastolic peak from the onset. S_peak_ = Amplitude of systolic peak from the onset.	AIx increases in older and CVD subjects (28).
PPG	Crest Time (CT)	*CT* = *Systolic*_*time*_ − *Onset*_*time*_	CT, Time from onset to systolic	CT is affected by aging and arteriosclerosis (29).
PPG	Crest Time Ratio (CTR)	$CTR=\frac{CT}{Cycle_{time}}$	CT, Crest Time; Cycle_Time_, Time from onset to next onset	CTR was lower in the non-diabetic subject than that in diabetic subjects (P = 0.004). Patients with diabetes have a higher risk of CVD (29).
APG	Ratio	$\frac{b}{a}$	Amplitude ratio	b/a increases as arterial stiffness increases (30).
APG	Ratio	$\frac{c}{a},\frac{d}{a},\frac{e}{a}$	Amplitude ratio	All ratios decrease as arterial stiffness increases (30).
APG	Vascular Aging (VA)	$VA=\frac{b-c-d-e}{a}\;$or $VA=\frac{b-e}{a}$	Aging Index	Assessment of vascular aging and arteriosclerotic disease (30).

## Methodology

Information on the derivatives marker discussed in the previous section is analyzed using the CSL Pulse Oximetry Artifact Labels database and hospital-acquired PPG data to develop complete peak detection of PPG and APG.

### PPG Data

There are a few available databases online that give a variety of PPG signals, such as CSL Pulse Oximetry Artifact Labels (31), MIT-BIH Polysomnographic database (SLP) (32), and MIMIC Database (MIMIC) from PhysioNet (33). SLP database has ECG, PPG, respiration, and EEG records of 18 subjects with sleep apnea syndrome. Annotation for PPG fiducial points is not given; instead, beat-by-beat annotation is given for the ECG signal. Previous studies have used the R-peak annotation to evaluate their algorithm for systolic peak detection. The MIMIC database has 121 of 10-min segment records of ECG, respiration, ABP, and PPG from 90 ICU patients. Systolic peak annotations are given, but they are only available for selected records.

In this study, the CSL database [initially published by Aboy et al. (34)] is used as it provides ECG, PPG, and respiration signals with clear ECG R-peak and PPG systolic peak annotations from 8-min recordings. However, there are no variations in the PPGs as it involved waveforms from 2 patients only (31). Thus, PPG data are collected from 43 elective ischemic heart disease (IHD) subjects, aged 53.4 ± 10.38 years, at Hospital Canselor Tuanku Muhriz (HCTM), Universiti Kebangsaan Malaysia. Simultaneous recordings of ECG and PPG are made in 10-min duration using Maxim MAX86150EVS module. Laboratory blood test results, 12-lead ECG output, and angiogram evidence are collected as additional information for further investigation. The data collection is approved by the Research Ethics Committee of Universiti Kebangsaan Malaysia (UKM PPI/111/8/JEP-2020-806), and informed consent has been obtained from all subjects.

To the best of our knowledge, the evaluations of complete fiducial point detection of PPG, VPG, or APG in previous studies are done manually by skilled medical persons. Currently, there is no expert on our side to validate the results for every fiducial point. However, with the evidence of R-peaks from the recorded ECG waveform, the systolic peaks of the PPG waveform of all 43 IHD subjects are manually determined. This has been possible since the simultaneous recording of ECG and PPG is made. R-peak of ECG is detected using the notable Pan-Tompkins method. An average of 1-min recording waveforms is manually selected for this purpose using the signal quality indexing method, as described in Nayan and Hamid (35).

### Derivative Marker

The derivatives of PPG up to the fourth level are calculated using Equations (1)–(4), where x(t) is a clean PPG signal, filtered using Chebyshev Type II and moving average filters. The first derivation of PPG produced VPG, whereas the second derivation produced APG, which reflects the velocity and acceleration of the blood, respectively. The third derivative is the acceleration rate or changes in acceleration over time. This is called “Jerk.” Changes of “Jerk” over time are “Snap.” Thus, the name JPG and SPG are proposed for the third and fourth PPG derivatives, respectively. This is the continuity of what has been proposed by Elgendi et al. (23). Fiducial points for each waveform are listed in Table 2.


(1) $$\begin{array}{l} VPG=\frac{d}{dt}\left(PPG\right)=\;\frac{d}{dt}\left[x\left(t\right)-x\left(t-1\right)\right] \end{array}$$

(2) $$\begin{array}{l} APG=\frac{d}{dt}\left(VPG\right)=\;\frac{d}{dt}\left[x\left(t\right)-2x\left(t-1\right)+x\left(t-2\right)\right] \end{array}$$

(3) $$JPG=\frac{d}{dt}(APG)=\;\frac{d}{dt}[x\left(t\right)-3x\left(t-1\right)+3x\left(t-2\right)-x(t-3)]$$

(4) $$SPG=\frac{d}{dt}\left(JPG\right)=\;\frac{d}{dt}[x\left(t\right)-4x\left(t-1\right)+6x\left(t-2\right)-4x\left(t-3\right)+x(t-4)]$$

**Table 2:** Fiducial points of PPG and its derivatives.

**Waveform**	**Abbr**.	**Fiducial Point**	**Label**
PPG	PPG	Onset	O
		Systolic peak	S
		Dicrotic notch	N
		Diastolic peak	D
First derivative PPG	VPG	Maximum peak in systole	*u*
		Minimum peak after *u*-peak at late systolic	*v*
		Maximum peak in diastole	*w*
Second derivative PPG	APG	Maximum peak in systole	a
		Minimum peak after a-peak	b
		First positive peak after b-peak at late systolic	c
		First negative peak after c-peak at late systolic	d
		Beginning of the diastolic component	e
		First negative peak after e	f
Third derivative PPG	JPG	Early systolic component	p0
		Middle systolic components	p1, p2
		End systolic component	p3
		Early diastolic component	p4
Fourth derivative PPG	SPG	Early systolic component	q1
		Middle-systolic components	q2, q3
		Early diastolic component	q4

**Figure 3:**
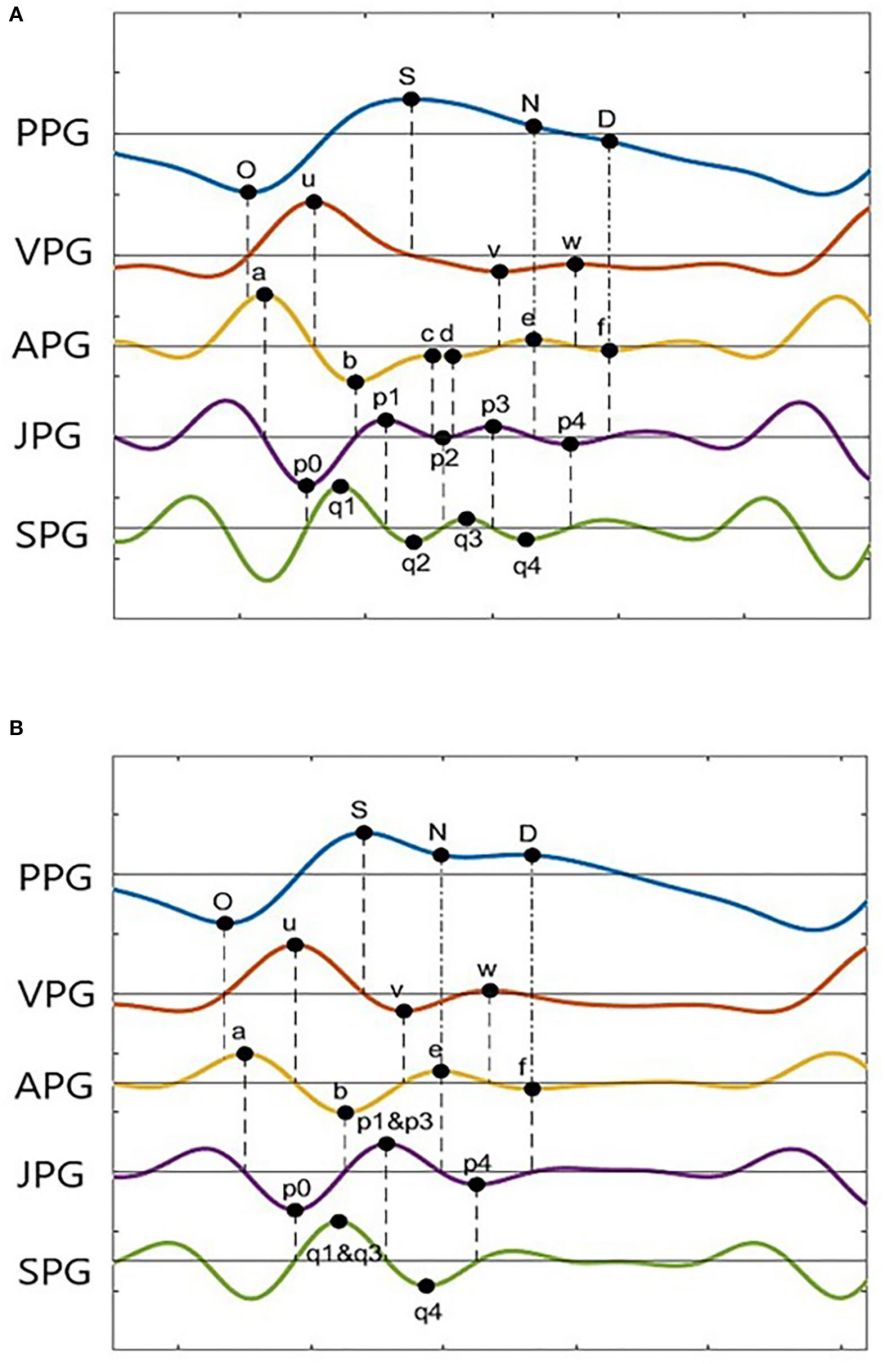
PPG derivatives marker from two typical morphologies of PPG **(A)** Type I, **(B)** Type II.

Photoplethysmogram and its derivative waveforms of the 43 IHD subjects are examined to see which points (derivative markers) are consistently intersecting between the derivatives and the original signal. Figure 3 shows the example of two common types of PPG and their derivatives found in our database (Type I and Type II). These waveforms have been normalized for better visualization. Type II waveform has clearer N-peak and D-peak compared to Type I waveform. However, more inflection points on PPG derivatives can be found in the Type I signal, which will be helpful in peak detection.

A derivative algorithm analyzes each curve's slope changes or turning point in a signal. For example, changes in PPG amplitude from O-peak to S-peak have produced the u-wave in VPG. Zero amplitude changes at O-peak give zero velocity in VPG. A constant increase in amplitude at the middle of O-peak and S-peak gives a constant velocity of u-peak, and zero amplitudes change at S-peak give zero velocity in the VPG. Thus, zero-crossing information of the u-wave of the VPG can be used to find the onset and systolic component.

In general, zero-crossing before a derivative peak represents the negative peak of its original signal, whereas zero-crossing after a derivative peak represents the positive peak of its original signal. Figure 3A shows 18 zero-crossing information-related derivative waves to their original signals. These are represented by the dashed line. The dash-dot line represents other markers as previously reported, where the e-peak of APG is equal to the N-peak of PPG, and the f-peak is the diastolic peak. There are frequent cases where the c-peak and d-peak of APG are missing or merged in e-waves. Consequently, the p1, p2, and p3 waves are merged in a single wave. The same goes for q1, q2, and q3 in SPG waveform. This is shown in Figure 3B. In certain subjects, w-wave can have a negative value, where zero-crossing points are unavailable. It will complicate the peak detection of the D-peak of PPG. Nevertheless, other derivative markers could be used to detect peaks and estimate their location or range of interest.

### Peak Detection

This study proposed a simple peak detection method named Derivative Marker Method (DMM). It focused on detecting four important PPG points, three VPG points, and five APG points, which are presently essential for feature extraction of the PPG signal. Pre-processing of PPG is required to get a clean waveform pattern (36). PPG signal is firstly filtered using fourth-order Chebyshev Type II filter with cutoff frequencies of 0.5 and 8 Hz. The filter is selected based on the recommendation by Liang et al. (37), who have appropriately assessed nine types of filters from 10 different orders for PPG (37). There might be a low-frequency respiration component included and a filtering effect that could contribute to baseline wander or dc-offsetting of the signal. These are removed by subtracting each sample of a PPG with its mean value. Derivatives of PPG are then calculated. After each derivation, the moving average filter is applied using Equation (5) to remove unnecessary small ripples that complicate the peak detection process. The n represents the number of samples, x[n] is the output of the moving average filter, y[n] is the input signal, and W is the window width of 50 ms.


(5) $$x[n]=\;\frac{1}{W}(y\left[n-\frac{W-1}{2}\right]+\ldots +y[n]+\ldots +y\left[n+\frac{W-1}{2}\right])$$

The first vital peak to be detected is the u-peak of VPG. The peak is dominant and typically higher in amplitudes compared to the w-peak. An amplitude threshold of 0.3 ^*^ max (VPG) is set with a minimum peak distance of 250 ms since there is less likely for four pulses (four u-peaks) to appear in 1 s in rest condition (HR=240 bpm). The O-peak and S-peak of the PPG are determined by finding the zero-crossing point before and after the u-peak, respectively. Similar to u-peak, p0 of JPG is dominant in the negative amplitude. A threshold of 0.3 ^*^ min (JPG) with a minimum peak distance of 250 ms is used to get a negative p0-peak. The a-peak and b-peak of APG are then determined using the zero-crossing information of the p0-wave in the third derivative waveform.

There are possibilities of merged peaks in APG, JPG, and SPG. The peaks of p1, p2, and p3 are better recognized using zero-crossing information of the SPG. Thus, the study proposed to determine q1-peak and q3-peak first. The q1-peak is the first positive peak after the p0-peak location. The second positive peak is q3-peak. Then, zero-crossing after q3-peak is p3-peak of JPG. Zero-crossing after the p3-peak is the e-peak. The e-peak then examines whether its location is bigger than 0.55 ^*^ current a-a interval. If yes, then the q1-q3 peaks and p1-p3 peaks are considered merged. The factor of 0.55 is chosen after a series of tests and trials on our dataset (subjects aged 32 years and above).

**Figure 4:**
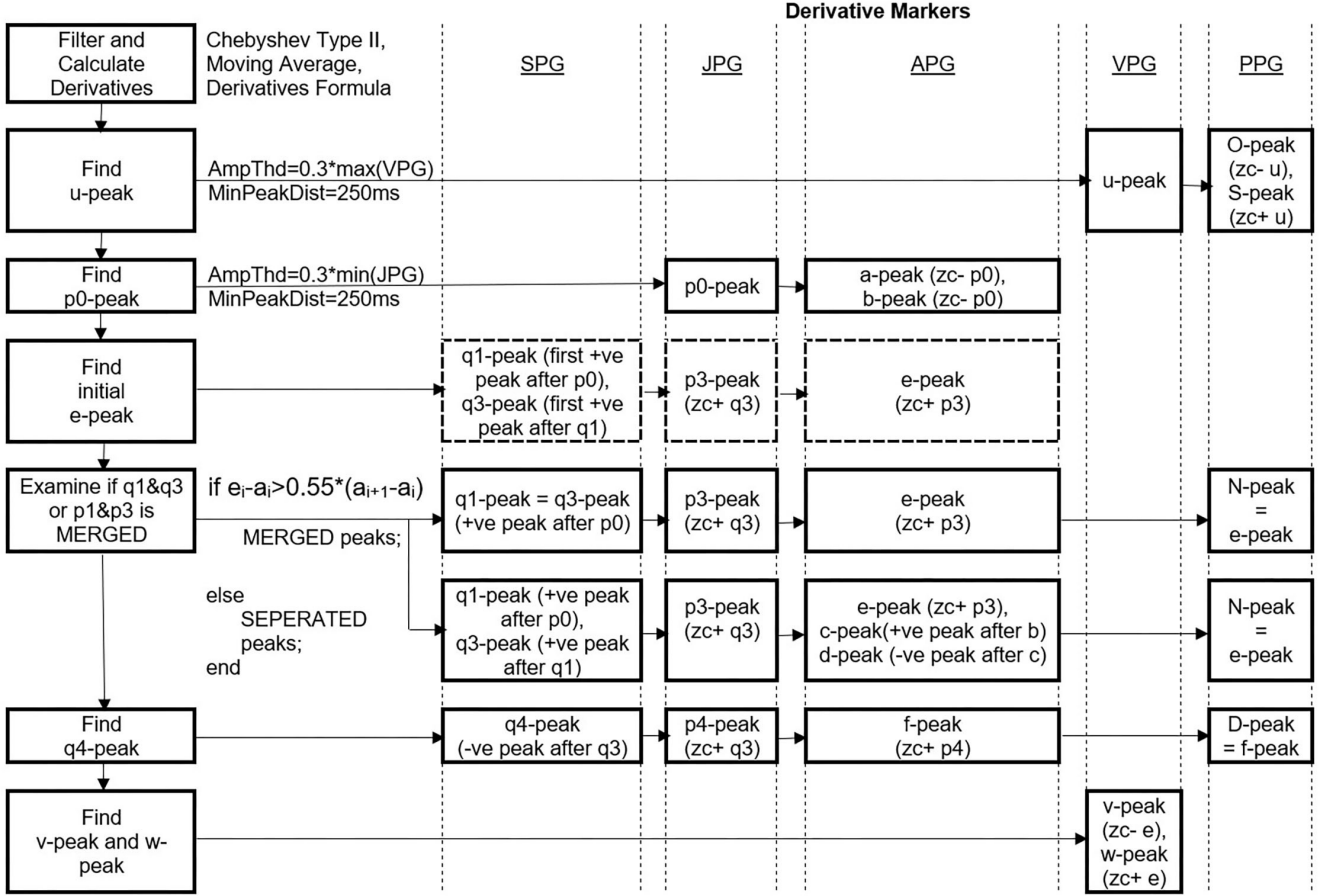
Peak detection steps using DMM. *Ampthd, Amplitude threshold; MinPeakDist, Minimum peak distance; zc-, zero-crossing before; zc+, zero-crossing after, –ve, negative, +ve, positive.

The q4-peak of SPG is determined afterward. It is the negative peak following the q3-peak. Zero-crossing after q4-peak is equal to p4-peak location, and zero-crossing after p4-peak is the f-peak in APG. Thus, the N-peak and D-peak of PPG are equal to the e-peak and f-peak of APG, respectively. There are cases where p2-wave has a positive value only. Thus, c-peak and d-peak cannot be determined using zero-crossing information. Therefore, the positive peak found between the b-peak and e-peak of APG is the c-peak, and the negative peak between the c-peak and e-peak is the d-peak. Meanwhile, the v-peak and w-peak of VPG are represented by zero-crossing before and after e-peak, respectively. The whole process is simplified as in Figure 4.

## Results and Discussion

At present, only systolic annotations are available for evaluation. A total of five performance matrices are calculated to evaluate the systolic peak detection using Equations (6)–(10), which includes sensitivity (SN), positive predictivity (PPV), accuracy (ACC), error rate (ERR), and mean absolute error (MAE). Complete results for systolic peak detection of the two CSL data and 43 IHD subjects' data are shown in Table 3.


(6) $$\begin{array}{l} SN=\frac{TP}{TP+FN}\times 100\% \end{array}$$

(7) $$\begin{array}{l} PPV=\frac{TP}{TP+FP}\times 100\% \end{array}$$

(8) $$\begin{array}{l} ACC=\frac{TP}{TP+FP+FN}\times 100\% \end{array}$$

(9) $$\begin{array}{l} ERR=\frac{FP+FN}{Total\;Beat}\times 100\% \end{array}$$

(10) $$MAE=\;\frac{1}{N}\sum_{i=1}^{N}\left|Det\_loc_{i}-Ann\_loc_{i}\right|$$

**Table 3:** Performance of systolic peak detection from two CSL subjects and 43 IHD subjects.

**Subjects ID**	**PPG Duration**	**Annotated Beat**	**Detected Beat**	**TP**	**FP**	**FN**	**SN (%)**	**PPV (%)**	**ERR (%)**	**ACC (%)**	**MAE (ms)**
CSL_009	8 min	816	815	815	0	1	99.88	100	0.12	99.88	000
CSL_015	8 min	960	959	959	0	1	99.90	100	0.10	99.90	000
IHD_04	60 s	81	81	81	0	0	100	100	0	100	000
IHD_06	60 s	85	85	85	0	0	100	100	0	100	000
IHD_07	60 s	99	99	99	0	0	100	100	0	100	000
IHD_10	60 s	44	44	44	0	0	100	100	0	100	000
IHD_12	60 s	57	57	57	0	0	100	100	0	100	000
IHD_13	60 s	79	79	79	0	0	100	100	0	100	000
IHD_16	60 s	59	59	59	0	0	100	100	0	100	013
IHD_21	60 s	57	57	51	6	0	100	89.47	10.53	89.47	0.0224
IHD_22	60 s	45	45	45	0	0	100	100	0	100	000
IHD_23	60 s	70	70	70	0	0	100	100	0	100	000
IHD_26	60 s	69	70	69	1	0	100	98.57	1.45	98.57	0.0123
IHD_27	60 s	54	54	53	1	0	100	98.15	1.85	98.15	038
IHD_28	60 s	79	79	79	0	0	100	100	0	100	000
IHD_31	60 s	69	68	68	0	1	98.55	100	1.45	98.55	000
IHD_33	60 s	54	54	54	0	0	100	100	0	100	000
IHD_34	60 s	62	62	61	1	0	100	98.39	1.61	98.39	040
IHD_35	60 s	81	81	81	0	0	100	100	0	100	000
IHD_36	60 s	75	75	73	2	0	100	97.33	2.67	97.33	083
IHD_37	60 s	78	78	78	0	0	100	100	0	100	0.0150
IHD_38	60 s	52	52	52	0	0	100	100	0	100	000
IHD_39	60 s	63	56	56	0	7	88.89	100	11.11	88.89	000
IHD_40	60 s	87	86	86	0	1	98.85	100	1.15	98.85	000
IHD_41	60 s	64	64	64	0	0	100	100	0	100	000
IHD_42	60 s	86	85	81	4	1	98.78	95.29	5.81	94.19	0.0149
IHD_44	60 s	59	59	59	0	0	100	100	0	100	000
IHD_45	60 s	65	65	64	1	0	100	98.46	1.54	98.46	056
IHD_46	60 s	59	59	59	0	0	100	100	0	100	000
IHD_47	60 s	69	69	69	0	0	100	100	0	100	000
IHD_48	30 s	35	36	35	1	0	100	97.22	2.86	97.22	094
IHD_49	60 s	57	57	57	0	0	100	100	0	100	000
IHD_52	10 s	7	7	7	0	0	100	100	0	100	000
IHD_53	60 s	80	80	80	0	0	100	100	0	100	000
IHD_55	60 s	68	68	68	0	0	100	100	0	100	000
IHD_56	60 s	68	68	68	0	0	100	100	0	100	000
IHD_57	60 s	90	90	90	0	0	100	100	0	100	000
IHD_58	60 s	58	58	58	0	0	100	100	0	100	000
IHD_60	60 s	70	70	70	0	0	100	100	0	100	000
IHD_61	60 s	48	48	48	0	0	100	100	0	100	000
IHD_62	60 s	61	61	61	0	0	100	100	0	100	000
IHD_63	60 s	64	64	64	0	0	100	100	0	100	0.0371
IHD_64	60 s	69	69	69	0	0	100	100	0	100	000
IHD_65	60 s	64	64	64	0	0	100	100	0	100	000
IHD_66	30 s	28	28	28	0	0	100	100	0	100	000
**Total**		**4,544**	**4,534**	**4,517**	**17**	**12**	**99.64**	**99.38**	**0.98**	**99.64**	**4.66 × 10** ^**−6**^
**performance**

where

TP = correctly detected peak (<=100 ms difference)

FP = incorrectly detected peak (>100 ms difference)

FN = missing peak (annotated beats – detected beats)

N = number of detected beats

Det_loc = detected location (in millisecond)

Ann_loc = annotated location (in millisecond)

**Figure 5:**
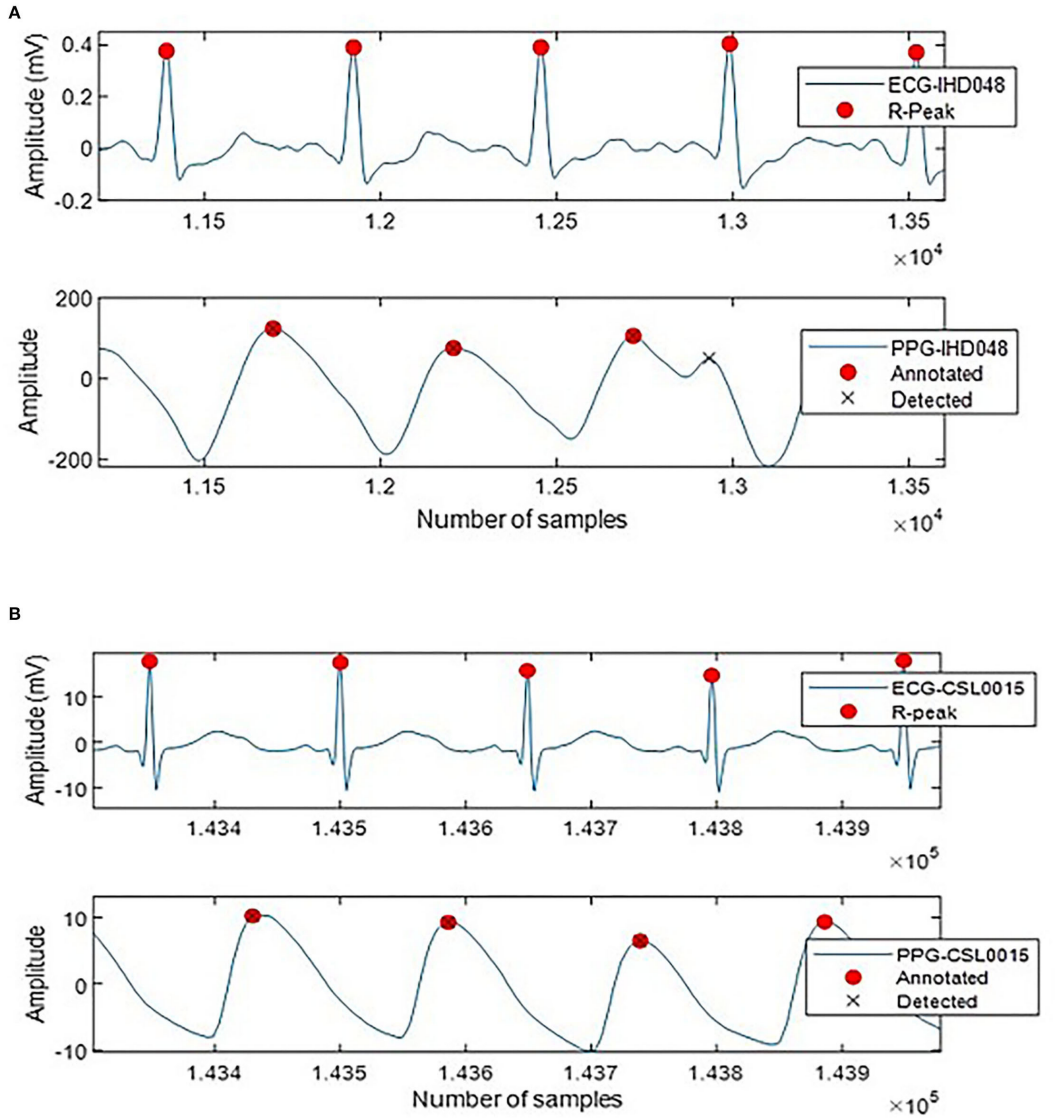
Annotated and detected systolic peak of **(A)** IHD048 with one false peak, **(B)** CSL0015 with one undetected peak.

A total of 4,517 out of 4,544 systolic peaks are correctly detected, with 17 false peaks and 12 missing peaks. Overall, the percentage of sensitivity, positive predictivity, and accuracy of the system are 99.64, 99.38, and 99.64%. The error rate is 0.98% with a very small MAE of 4.66 × 10^(^−6) ms. Figure 5 shows the examples of false detected peaks and undetected peaks from two PPG waveforms of CSL0015 and IHD048 subjects.

Table 4 compares SN and PPV for systolic peak detection between DMM and previous studies. A direct comparison cannot be made since most studies used different datasets and different medical experts to manually annotate the systolic peaks. In general, the proposed method has achieved significant SN and PPV of 99.64 and 99.38%, respectively. The result is comparable with Chakraborty et al. (12) and Ferro et al. (18), which used a complex algorithm involving the Hilbert transform.

**Table 4:** Comparison of systolic peak detection of DMM with other existing techniques.

**References**	**Data**	**Method**	**Beat**	**SN**	**PPV**
Aboy et al., 2005	CSL database	The rank order of bandpass filters and decision logic	42539	99.36	98.43
Shin et al., 2009	18 young & healthy subjects	Adaptive thresholding (ADT)	22622	98.04	100
Li et al., 2009	Fantasia database & SLP database	ABP waveform delineator	2564	99.88	99.45
Elgendi et al., 2013	40 healthy subjects	Event-related moving average filter & thresholding	5071	99.84	99.89
Ferro et al., 2015	10 volunteers	Shannon energy envelope, zero-phase filtering, and Hibert transform	2286	100	100
Vadrevu and Manikandan, 2016	CSL database	Variational Mode Decomposition (VMD) & Center of Gravity (COG)	12702	99.36	98.43
Paradkar et al., 2015	CSL database	Singular Value Decomposition (SVD) & wavelet	13079	99.13	99.84
Argüello Prada et al., 2019	8 young & healthy subjects	Mountaineer's method	7483	98.68	98.26
Chakraborty et al., 2020	MIMIC database & volunteers from healthy and CVD subjects	Signal derivative, Hilbert Transform on APG	17442	99.98	100
Proposed method, 2022	CSL database and 43 IHD subjects	Derivatives marker method (DMM)	4544	99.64	99.38

**Figure 6:**
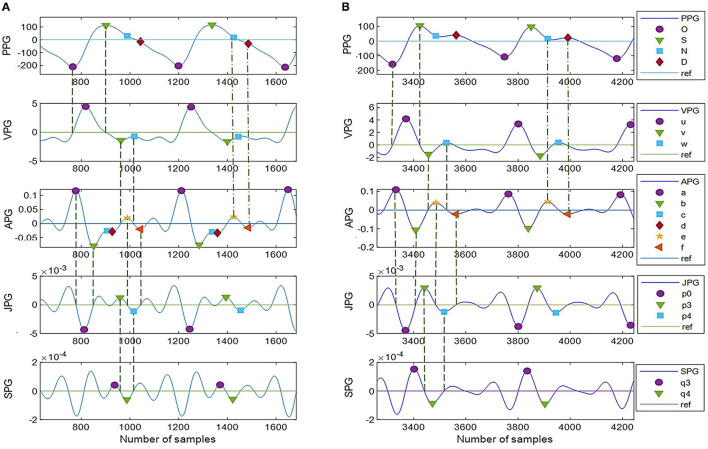
**(A,B)** Peak detection using DMM with twelve derivative markers. Type I: unclear N-peak and D-peak but with more inflection points on derivative waveforms, Type II: clear N-peak and D-peak but less information on derivative waveforms.

Figure 6 shows complete PPG and APG peaks detected on two common types of PPG signals using the proposed DMM. In Figure 6A, the notch and diastolic peaks in PPG are ambiguous, but it is apparent in the derivatives. Meanwhile, in Figure 6B, c-peak and e-peak are merged, but DMM can still locate e-peak with the information obtained in JPG and SPG.

To the best of our knowledge, this is the first study that analyzes PPG derivatives up to the fourth level. A total of twelve out of twenty derivative markers discovered are found useful for peak detection of two common types of PPG. The proposed DMM is quite simple. It uses a simple thresholding method to locate the dominant peak (u-peak of VPG and p0-peak of JPG) without back-searching or adjusting the threshold. However, good quality PPG recordings or proper filtering techniques are substantially needed. Moving average filters after each derivation is still required in the presently proposed techniques, which might cause losing information in the derivative curve patterns. SPG waveforms with four fiducial points of q1, q2, q3, and q4 are introduced in this paper. These points can be clinically explored in future disease prediction or classification studies.

## Conclusion

A total of four levels of PPG derivatives have been analyzed, and 12 derivative markers are discovered useful for complete fiducial point detections. The proposed DMM method is quite straightforward as it used derivative markers, moving average filter, and simple thresholding technique, without the need for a complex mathematical algorithm. It can adapt to different PPG morphologies of the merged waves in the derivative signal. However, the evaluation is limited to systolic peak detection only. Comparable sensitivity and positive predictivity of systolic peak detection of more than 99% have been obtained.

## Data Availability Statement

Publicly available datasets were analyzed in this study. This data can be found here: https://dataverse.scholarsportal.info/dataset.xhtml?persistentId=10.5683/SP2/SJAKCB.

## Ethics Statement

The studies involving human participants were reviewed and approved by Research Ethics Committee of Universiti Kebangsaan Malaysia (UKM PPI/111/8/JEP-2020-806). The patients/participants provided their written informed consent to participate in this study.

## Author Contributions

All authors listed have made a substantial, direct, and intellectual contribution to the work and approved it for publication.

## Funding

This work was supported by the research grant from the Ministry of Higher Education Malaysia (Grant No: TRGS/1/2019/UKM/01/4/3).

## Conflict of Interest

The authors declare that the research was conducted in the absence of any commercial or financial relationships that could be construed as a potential conflict of interest.

## Publisher's Note

All claims expressed in this article are solely those of the authors and do not necessarily represent those of their affiliated organizations, or those of the publisher, the editors and the reviewers. Any product that may be evaluated in this article, or claim that may be made by its manufacturer, is not guaranteed or endorsed by the publisher.
